# Determinants of Visceral Leishmaniasis in Addis Zemen Health Center, Northwest Ethiopia

**DOI:** 10.1155/jotm/5554577

**Published:** 2024-11-26

**Authors:** Atsedemariam Nigus Gedamu, Asrat Atsedeweyn Andargie, Aragaw Eshetie Aguade, Samuel Derso Tezera

**Affiliations:** ^1^Department of Epidemiology and Biostatistics, College of Medicine and Health Sciences, Institute of Public Health, University of Gondar, Gondar, Ethiopia; ^2^Department of Statistics, College of Natural and Computational Science, University of Gondar, Gondar, Ethiopia; ^3^Department of Veterinary Clinical Medicine, College of Veterinary Medicine and Animal Sciences, University of Gondar, Gondar, Ethiopia

## Abstract

**Background:** Visceral leishmaniasis (VL) is one of the public health issues in some areas of Ethiopia, and over 3.2 million people are at risk with an estimated 4000 new cases occurring each year in the country. This study was conducted to determine the prevalence of VL and its associated risk factors in Addis Zemen Health Center, Northwest Ethiopia.

**Methods:** Data were collected from Addis Zemen Health Center and meteorological office in Addis Ababa from 2012 to 2016. Univariate and multivariate analyses were conducted to identify the determinants of VL. According to the result obtained from the retrospective data analysis, a total of 4100 suspected VL patients diagnosed by rk39 in Addis Zemen Health Center from Libokemkem and nearby districts.

**Results:** The overall prevalence of VL among study participants were 30% (1230/4100). Of this, the prevalence of VL among male and female study participants was 86.8% and 13.2%, respectively. The proportion of sex infected by VL was 3.26 times higher in male than female (AOR = 3.26, 95% CI: 2.42–4.40). The risk of acquiring VL in those people living in rural area was 62% more likely than those residing in urban (AOR = 1.62, 95% CI: 1.29–2.04). People that were traveled to the endemic area of VL were 18.44 times more likely to be affected than the people who have not traveled once (AOR = : 18.44, 95% CI: 14.49–23.47). Age, sex, residence, season, travel history to endemic areas, and mean monthly precipitation were found to be statistically significant for VL at 5% significance level.

**Conclusion and Recommendation:** The prevalence of VL in the present study was high with the highest prevalence in the rural areas. Therefore, there is a need of the immediate establishment of sound control and prevention program in rural areas.

## 1. Introduction

Leishmaniasis is caused by protozoan Leishmania parasites, an obligate intracellular pathogen that invades phagocytic host cells, which is transmitted through female sandfly bites either zoonotically or anthroponotically, and more than 98 nations have reported the disease in the tropics and semitropic areas [1, 2]. Leishmaniasis is one of the most serious neglected tropical disease that mainly affect poor people residing in the Americas, Southeast Asia, East Africa, and the Mediterranean Basin with limited resources for treatment [2]. Though there are many distinct clinical presentations of leishmaniasis, cutaneous, mucosal, and visceral leishmaniasis (VL) are the three primary phenotypic types of the illness [3].

VL is one of a parasitic disease caused by Leishmania donovani complex, infiltrates the spleen, liver, bone marrow, and lymph nodes, resulting in pancytopenia and organomegaly, which, if untreated, is almost invariably fatal and severe form of leishmaniasis [4]. With the exception of Australia and Antarctica, VL has been reported on every continent and six nations such as India, Bangladesh, Brazil, Sudan, South Suda, and Ethiopia account for more than 90% of all cases worldwide [5]. Leishmania donovani is common in Asia and Eastern Africa, whereas Leishmania infantum is in Latin America and Mediterranean area [6].

VL is one of the public health issues in some areas of Ethiopia, and over 3.2 million people are at risk with an estimated 4000 new cases occurring each year in the country. VL was formerly referred to as the diseases of the lowlanders in lower and upper kola agro ecological zones of Ethiopia. VL is no longer an issue only affecting lowlanders, as seen by the 2005 upto 2007 outbreaks in the highlands of Libo Kemekem and Fogera, in the Woina Degas, which killed hundreds of people after being mistakenly diagnosed with drug-resistant malaria. The Metema-Humera focus is a major VL focus and accounts for approximately 60% of the total disease burden in Ethiopia [7].

A case-control study was carried out during the period of January–July 2013 in Tigray, northwest Ethiopia with the aim of identifying the factors associated with VL using a binary logistic regression model, and he found that most of the cases (825) were in the age group 15–39 years, the second affected age group were 5–14 years followed by < 5 years and > 40 years [8].

A study carried out by authors in [9] using the chi-square (*X*^2^) test to determine the seven year–trend prevalence of VL in Addis Zemen Health Center, northwest Ethiopia, from 2005 to 2011 using a univariate analysis, majority 2624 (39.5%), of the VL confirmed cases were from the rural area. This may be associated with sandfly breeding sites, and it is assumed that the breeding sites are found more concentrated in the rural areas than in urban environments.

In addition, knowing and updating epidemiology of VL in a particular place is crucial. Thus, investigating determinants of VL has paramount importance to develop effective prevention and control strategies to reduce its prevalence and provide information to policy maker and stakeholders to take intervention actions towards key determinant factors of VL. Therefore, the study was aimed to determine the prevalence and assessing its determinants of VL in Addis Zemen Health Center, northwest Ethiopia.

## 2. Materials and Methods

### 2.1. Study Area

Libo Kemekem district is 2000 m above sea level and situated in the South Gondar administration zone in the Amhara region of northwest Ethiopia. The estimated population of the district, which consists of 30 kebeles, is 198,374. Addis Zemen is located approximately 637 km away from Ethiopia's capital with the population 19,755. In addition to Libo Kemekem district, the health center covers neighboring areas such as Fogera, which is projected to have a population of 2,26,595.

### 2.2. Study Design and Period

A retrospective study was conducted from Addis Zemen Health Center from October, 2012, to June, 2016, to determine the prevalence and its associated risk factors of VL. Data about monthly meteorological variables including mean maximum and minimum temperature, and precipitation was extracted from Meteorological office in Addis Ababa from 2012 to 2016.

### 2.3. Variables Considered

#### 2.3.1. Dependent Variable

The response variable is “Prevalence of VL” in Addis Zemen Health Center which is binary with two outcomes “positive” or “negative.”

#### 2.3.2. Independent Variable

Climate variables such as monthly mean maximum and minimum temperatures, and monthly mean amount of precipitation and other nonclimatic variables such as as age, sex, residence, season, and travel history to endemic areas such as Metemma and Humera districts were independent variables.

### 2.4. Sample Size and Sampling Procedures

Using a temporary worksheet with the necessary information, data were manually collected from the laboratory registration book that have VL direct agglutination test (DAT)/rk39 results by using a convenience sampling technique, and a total of 4100 VL suspected cases were found in the record and considered as study participants.

### 2.5. Data Management and Analysis

Prior to the statistical analysis, the collected data were coded correctly, checked for errors, and entered and saved into a Microsoft Excel spreadsheet 2010, and the imported data were analyzed by Statistical Package for Social Sciences (SPSS) software version 20.0. Descriptive statistics were used to show the occurrence of VL year wise and with different independent variables. The associations of the VL based on the different risk factors such as age, sex, residence, and season, travel history to endemic area, maximum, minimum temperature, and precipitation were assessed by using univariate and multivariate logistic regression with 95% confidence interval and less than 0.05 level of precision.

## 3. Results

The main purpose of this chapter is to describe results of descriptive statistics, univariate and multivariate analyses by using binary logistic regression. According to the result obtained from the retrospective data analysis, a total of 4100 VL suspected patients diagnosed to Addis Zemen Kaalazar/Health Center from Libokemkem and nearby districts. Out of this, 1230 (30%) were positive for VL by rk39 kit/DAT and the rest 2870 (70%) were negative. The highest prevalence were recorded in 2012, 34.2% (294/859), followed by 2013, 30.9% (362/1173), 2014, 30.8% (260/844), 2016, 29.3% (148\505), and 2015, 23.1% (166/719). Of all, 3557 (86.8%) were males and 543 (13.2%) were females and 3436 (83.8%) reside in rural and 664 (16.2%) were from urban. Of this, the prevalence of VL among male and female study participants was 32.6% (1160/3557) and 12.9% (70/543), respectively. From the total VL confirmed cases, 32% (1098/3436) in the rural areas and 19.9% (132/664) were from urban areas. The prevalence of VL in age groups 39 years was 26.5%, 30.1%, 31.4%, and 21.2%, respectively. The highest prevalence of VL cases was observed in dry season (33.3%) followed by long rainy (27.8%), and short rainy season (27.8%) are indicated in Table 1. The prevalence among study participants who had history of traveling to the endemic area was 83.2% and 21.4% to those who do not have history of traveling.

The estimated odds ratio 3.07 (OR = 3.07, 95% CI: 1.90–4.95) indicates that the age between five and fifteen are 3.07 times more likely to get VL compared to those below 5 years age group controlling for other variables constant in the model. The estimated odds ratio 1.99 (OR = 1.99, 95% CI: 1.37–2.89) indicates that age between fifteen and 39 years of age are 99% more likely to have VL compared to the reference group “below 5 years” age group controlling for other variables constant in the model. The risk of having VL in age group above 39 were 58% (OR = 1.58, 95% CI: 1.22–2.06) more likely compared to below 5 years age group controlling for other variables constant in the model.

The proportion of sex infected by VL was 3.26 times higher in male than female (OR = 3.26, 95% CI: 2.42–4.40) controlling for other variables constant in the model. The risk of acquiring VL in those people living in the rural area was 62.4% more likely than those residing in urban (reference group) (OR = 1.624, 95% CI: 1.29–2.04) controlling for other variables constant in the model. People that had traveled to the endemic area of VL were 18.44 times more likely to be affected than the people who have not traveled once (OR = 18.447, 95% CI: 14.49–23.47) controlling for other variables in the model. The risk of having VL was 30% more likely to occur in the dry season than after long rainy season (OR = 1.30, 95% CI: 1.04–1.63). The odds of having VL were increased by 2% for a unit change in precipitation. Age, sex, residence, season, travel history to endemic areas, and precipitation were significantly (*p* < 0.05) influenced by the occurrence of VL in the current study.

**Figure 1:**
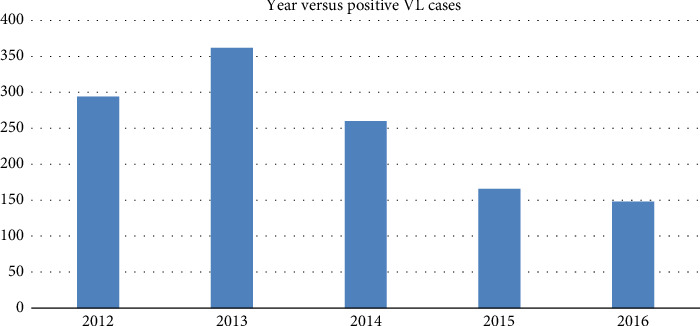
VL cases across different years of diagnosis.

In relation to the years, the highest number of VL cases was recorded in 2013 (*n* = 362) followed by 2012 (*n* = 294), 2014 (*n* = 260), 2015 (*n* = 166), and 2016(*n* = 148) as indicated in Figure 1.

**Figure 2:**
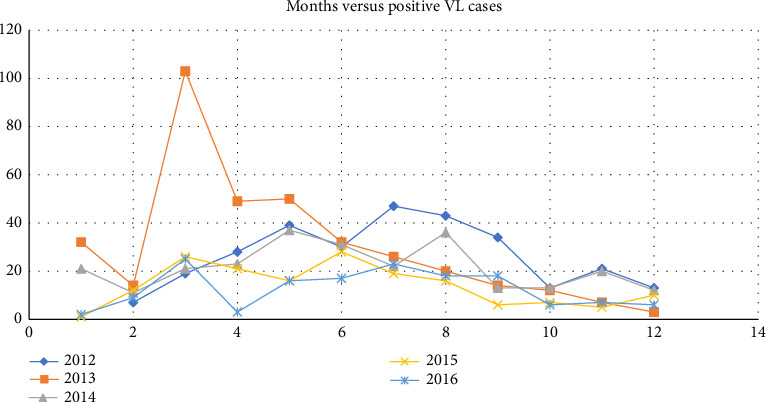
Plot showing seasonal occurrence of VL in Addis Zemen Health Center during the period of 2012–2016.

In line with the different seasons of the year, the highest overall frequency of VL cases was observed during November to May in all of the 5 years (Figure 2).

## 4. Discussion

The overall prevalence of VL among study participants were 30% (1230/4100) in the study area which is in agreement with the study carried out by authors in [10] with 32.4% prevalence of VL in Metemma and Addis Zemen Hospitals. On the hand, higher prevalence were reported by authors in [9] with the prevalence of 39.1% in Addis Zemen Health Center. This difference in prevalence might be due to the awareness creation about the control and prevention strategies of VL for the migrant labor workers who traveled to the endemic areas.

In the present finding, the prevalence of VL in rural and urban area was 32% and 19.9%, respectively, with a statically significant result. People living in the rural area are more exposed to VL as compared to urban. This finding is in line with a study carried out in Ethiopia by authors in [9] in which rural areas accounted for the majority of the 2624 (39.5%) VL-confirmed cases. The reason for this could be that sandfly breeding grounds are more common in rural than in urban settings [11].

In the current study, the prevalence of VL among male and female study participants was 32.6% (1160/3557) and 12.89% (70/543), respectively. This finding is in agreement with study carried out in Ethiopia by authors in [8, 11, 12]. This gender difference might be due to males are more involved in outdoor activities such as agricultural tasks than females, that is why males are more vulnerable than females [13].

The risk of having VL in age group between 5 and 15 years of age is 3.07 times more likely compared to below 5 years of age with the prevalence of 30.1%, 26.5% in 5–15 years, and <5 years, respectively. This finding agrees with studies carried out in Ethiopia by authors in [9], whereby the majority of VL-confirmed cases (50.1%) were seen in the 5–14 years age range. A similar study carried out by authors in [9] showed most positive conversions occurring in the 10 to 20-year-olds. This might be due to young study participants are expected to keep domestic animals outdoors especially during dawns in the study area, and this exposed them to the bite of sandflies. The result indicates that age between fifteen and 39 years are 99% more likely to have VL compared to the reference group “below 5 years.” This finding is in line with the authors in [14] in which the degree of positivity was strongly correlated with increasing age. This finding is also in line with the authors in [11] who indicated that adult human beings older than 15 years were more affected than the lower age groups. A similar study in Ethiopia by authors in [10] also found that age was the most important determinant of VL.

In the current finding, travel to endemic areas was found as an important determinant for VL with a significant association. The prevalence among study participants who had history of travel to the endemic area was 83.2% and 21.4% to those who do not have history of travel. This indicates migration to the endemic area will contribute for having higher prevalence than nonendemic ones.

Out of the total, the highest prevalence were recorded in 2012 (34.2%) followed by 2013 (30.9%), 2014 (30.8%), 2016 (29.3%), and 2015 (23.1%). There was also a dramatic decrease in the number of VL cases detected from 2012 to 2016 due to early diagnosis and treatment of VL epidemic cases. This finding is also in line with a study carried out by authors in [11, 15, 16] who founded host factors, environmental, and labor migrants to the endemic areas contributing to epidemic.

In this finding, the odds of having VL were increased by 2.6% for a unit change in precipitation with a statistically significant result. This study is line with the study carried out in Ethiopia by the authors in [7] and also by authors in [16] in Montenegro in southeastern Europe, wherein they discovered a substantial correlation between the frequency of leishmaniasis cases in January, February, June, and December with each 1 mm rise in precipitation (mm). This could be because leishmaniasis is a climate-sensitive illness, in which weather factors such as precipitation have a significant influence on the behavior and reproduction of the sandfly, which is the disease's vector [16].

In the current study, the highest overall frequency of VL cases was observed during November to May in all of the 5 years, 2012–2016. This finding is line with the study carried out at Kahsay Abera Hospital, northwest Ethiopia, in which VL case load in the hospital peaked during dry season, January and February [8]. In addition, in this finding, the odds of having VL was 30% more likely to occur in the dry season than after long rainy season. This finding is in consistent with [8]. This might be due to high sandfly population which is a vector for the transmission of the VL during the dry season.

### 4.1. Limitations of the Study

The environmental and some sociodemographic risk factors that predispose peoples to VL were assessed. However, nutritional, HIV status, dog ownership, and socioeconomic status were not assessed due to the nature of the retrospective study.

## 5. Conclusion

Place of residence, age, sex, season, travel history to endemic area, season, and precipitation have a significant effect on VL occurrence in the study area. The findings of this study provide a useful and potentially valuable tool for VL control. In general, our findings showed that the prevalence of VL was significant with highest prevalence, in male, rural residence, 15–39-year age group, and during dry season. The finding of this study confirms that rural areas are more conducive for VL as compared to urban. Thus, it is recommended that control and prevention strategies should be made more in rural areas than in urban, and special attention should be given for males and 15–39 years age group who had more history of travel to endemic areas to minimize the prevalence of VL by taking into consideration the burden of VL in these groups.

## Figures and Tables

**Table 1:** Univariable and multivariable logistic regression output for the risk factors associated with VL.

Variable	Categories	VL		COR (CI: 95%)	AOR (CI: 95%)	*p* value
		Number examined	Number positive			
Age	< 5	113	30 (26.5%)	1	1	
	5–15	296	89 (30.1%)	4.22 (2.23–5.93)	3.07 (1.90–4.95)	≤ 0.001
	15–39	3205	1008 (31.4%)	3.13 (1.68–3.72)	1.99 (1.37–2.89)	≤ 0.001
	> 39	486	103 (21.2%)	2.05 (1.91–2.92)	1.58 (1.22–2.06)	0.001

Sex	Female	543	70 (12.9%)	1	1	
	Male	3557	1160 (32.6/%)	3.72 (2.73–4.80)	3.26 (2.42–4.40)	≤ 0.001

Residence	Urban	664	132 (19.9%)	1	1	
	Rural	3436	1098 (32%)	1.83 (1.47–2.27)	1.62 (1.29–2.04)	≤ 0.001

Season	After long rainy	1227	341 (27.8%)	1	1	
	Dry	1656	551 (33.3%)	1.94 (1.62–2.02)	1.30 (1.04–1.63)	0.018
	After short rain	1217	338 (27.8%)	2.31 (0.97–1.72)	1.14 (0.92–1.41)	0.205

Travel history to endemic area	No	3530	756 (21.4%)	1	1	
	Yes	570	474 (83.2%)	18.92 (14.89–23.87)	18.44 (14.44–23.47)	≤ 0.001

Maximum temperature (°C)	29.26	4100	1230 (30%)	1.58 (0.98–1.42)	0.99 (0.96–1.02)	0.504

Minimum temperature (°C)	12.46	4100	1230 (30%)	1.621 (0.996–1.671)	1.04 (0.98–1.10)	0.133

Precipitation (mm)	3.07	4100	1, 230 (30%)	1.591 (1.431–1.531)	1.02 (1.00–1.04)	0.010

## Data Availability

Data will be available from the corresponding author upon request.
